# Virulence Genotyping and Multidrug Resistance Pattern of Escherichia coli Isolated From Community-Acquired and Hospital-Acquired Urinary Tract Infections

**DOI:** 10.7759/cureus.29404

**Published:** 2022-09-21

**Authors:** Shruti Radera, Sugandha Srivastava, Jyotsana Agarwal

**Affiliations:** 1 Microbiology, King George's Medical University, Lucknow, IND; 2 Microbiology, Dr. Ram Manohar Lohia Institute of Medical Sciences, Lucknow, IND

**Keywords:** community-acquired uti, hospital-acquired uti, virulence factors, multidrug resistant, uti

## Abstract

Introduction

Uropathogenic *Escherichia coli*(UPEC) strains consist of a plethora of putative virulence factors (VFs), which help them to establish infection in the urinary tract. We compared genotypic profiles of *Escherichia coli (E. coli)* strains associated with community-acquired (CA) urinary tract infection (UTI; n=100) and hospital-acquired (HA) UTI (n=50) in the present study in order to identify specific virulence determinants, if any, associated with either form of UTI and its association with antibiotic resistance pattern of the isolates.

Materials and methods

*E. coli* strains were analyzed for antimicrobial susceptibility patterns, phylogroups, and 10 putative virulence-associated genes. The bacterial culture and identification were done using standard conventional methods. Tests for antimicrobial susceptibility and phenotypic detection for extende- spectrum beta-lactamases (ESBL) were done by using the Kirby Bauer disc diffusion method, and results were interpreted as per Clinical & Laboratory Standards Institute (CLSI) guidelines. The phylotype (A, B1, B2, and D) of each *E. coli* isolate was determined by a triplex polymerase chain reaction (PCR) based phylotyping method. They were further analyzed for the presence of 10 putative virulence genes (VGs), including adhesins papA (P fimbrial structural subunit), papG alleles I, II (P fimbrial adhesin variants), fimH (type 1 fimbriae), toxins hlyA (hemolysin) siderophores chuA (heme-binding protein); yfcV (encodes a major subunit of a putative chaperone-usher fimbria) capsule synthesis specific for group II (K1, K5, K12, etc.) kpsMII; serum resistance‐associated traT, and upaH by multiplex PCR.

Results

HA *E. coli* isolates were significantly more drug-resistant than CA isolates; carbapenem (80% vs. 16%), ceftazidime (92% vs. 63%). The majority (52%) of *E.coli* isolates associated with HA UTI belong to commensal phylogroup A and B1, whereas the majority (66%) in CA were from pathotypic phylogroups, i.e., B2 & D. Most of VFs were frequently present amongst CA group except for traT and yfc, kpsMTII, hlyA, chuA, and upaH were significantly associated with CA *E.coli* isolates while yfc was significantly present in HA *E.coli* isolates. Though adhesin genes such as papA, papGI, papGII, fimH were frequently found in the CA group, they were not significantly associated. The average virulence score was higher for CA UTI isolates (4.25) than for the HA strains (3.9). Multidrug resistance (MDR) was present in every HA *E.coli* isolate, and fimH, traT, and yfc genes showed significant association with MDR strains.

Conclusion

On detailed analysis, we found that HA *E. coli* isolates had a high frequency of MDR and comparatively reduced VFs content. Thus, it can be assumed that a strain with lesser virulence is able to cause HA UTIs, as compared to CA UTIs, which probably indicates that the host’s immune status/general condition can be an important determinant in acquiring infection rather than virulence potential of the pathogen alone.

## Introduction

Urinary tract infection (UTI) is one of the most common bacterial infections, mostly affecting young women and women in the early postmenopausal period [1]. UTIs represent a major public health burden worldwide [2, 3]. *Escherichia coli* (*E. coli*) is the single most important causative agent of UTIs, accounting for 75-85% of the episodes [4]. Though *E. coli* occurs as commensal in the gastrointestinal tract, few species cause intestinal and extraintestinal infection by virtue of possession of some specific virulence factors [5,6]. Uropathogenic *Escherichia coli* (UPEC) causes almost 90% of community-acquired (CA) UTIs and 50% of hospital-acquired (HA) UTIs. UPEC strains consist of a plethora of putative virulence factors (VFs) such as adhesins, toxins, siderophores, and capsules so as to establish an infection [7, 8, 9]. Treatment for uncomplicated CA UTI is done empirically most of the time and does not include obtaining a urine specimen for culture. Only limited laboratory-based data on resistant UPEC causing CA UTIs is available; furthermore, this does not include detailed molecular characterization of the isolates [10]. It has been estimated that catheter-associated UTIs are one of the most common causes of nosocomial infection and are most often caused by multidrug-resistant bacteria. In healthcare settings, most patients are immuno-compromised; many of them have indwelling urinary catheters and are being treated by a plethora of antimicrobial agents. These patients are at greater risk of developing UTIs by *E. coli *strains that are not considered typical uropathogens [11]. We can hypothesize that nosocomial UTIs are not caused by typical UPEC, but they may be caused by *E. coli* strains with unusual VFs groups. It has also been observed that there is an exchange of various virulence gene sets between *E. coli* [11]. As details of resistance patterns and molecular characterizations of UPEC are not much available, any potential bias towards the over-presentation of a clinical situation only on the basis of limited laboratory data should be avoided. Thus, it is imperative to study the detailed molecular characterization of the population structure of UPEC obtained from hospital and community-acquired UTIs. Several studies have shown that virulence factors do not act individually but in a coordinated way to guarantee the successful survival and persistence of UPEC in the hostile environment of the urinary tract [12-15].

Thus, the main aim of this study was to describe the molecular characteristics of the UPEC isolates collected from patients suffering from UTIs during inpatient or outpatient treatment at a tertiary care hospital. The isolates were studied by multiplex polymerase chain reaction (PCR) in order to detect, examine, and compare the possible virulence factor repertoire exhibited by *E. coli *strains isolated from community-acquired and hospital-acquired UTIs. In addition, antibiotic susceptibility profile and any correlation of VFs with antibiotic resistance, if present, were also studied.

## Materials and methods

Urine specimens of patients who presented with symptoms of UTIs, in both outpatient and inpatient departments, were included in the study. The present study was conducted between June 2016 to May 2018 in the department of Microbiology at King George's Medical University (KGMU), Lucknow, which is a tertiary care health center in Northern India. The study protocol was approved by the Institutional Review Board of KGMU.

Community-acquired UTI 

Sexually active women between the ages of 18 and 50 years with two or more symptoms suggestive of acute cystitis, including dysuria, urine frequency of > six times per day, urgency, haematuria/smoky urine, burning sensation during micturition, and acute onset incontinence consenting to participate and provide a mid‑stream clean catch urine samples were invited to take part in the study. Exclusion criterion includes no history of UTI in the last one year with bacteriologically documented *E. coli*, no underlying comorbidity, apparent urological abnormality, or a urethral catheter in place.

Hospital-acquired UTI

Patients not admitted with primary complaints suggestive of UTIs, who have bacteriologically documented *E. coli* positive urine sample which first appears after 48 hours of the patient's hospital admission, were considered as cases of HA UTIs [16].

Sample processing

Urine samples were immediately processed after collection and semi‐quantitatively cultured onto a cystine lactose electrolyte deficient agar plate (HiMedia, Mumbai, India). Lactose‐fermenting colonies with appropriate colony morphology were presumptively identified as *E. coli* and were further confirmed using standard conventional biochemical tests [17]. A colony count of >10^2^ CFU/ml was taken as significant growth [18].

Antibiotic susceptibility testing

Antimicrobial susceptibility testing for ampicillin, amoxyclav, norfloxacin, ciprofloxacin, nitrofurantoin, co-trimoxazole, gentamicin, amikacin, cefoxitin, aztreonam, ceftazidime, cefotaxime, meropenem, imipenem, and fosfomycin was performed using Kirby Bauer's disk diffusion method, and the results were interpreted as specified by CLSI guidelines [19]. Bacteria were classified into multi-drug resistant (MDR, resistant to at least one agent in three or more antimicrobial classes), extensively drug-resistant (XDR, non-susceptible to ≥ 1 agent in all but ≤ two categories); pan-drug resistant (PDR, not susceptible to all kinds of antimicrobial agents listed) [20].

Phenotypic detection of extended spectrum of β-lactamases

All the strains which tested resistant to cefotaxime or ceftazidime were subjected for the detection of an extended spectrum of β-lactamases (ESBL) by performing the combination disc method. Ceftazidime or cefotaxime disc alone and in combination with clavulanic acid (10μg) were placed on Mueller-Hinton agar plate seeded with 0.5 McFarland suspension of test strain. Increase of ≥5 cm zone of inhibition single disk and in combination with clavulanic acid was considered positive for an ESBL producer [21].

Phylogenetic grouping

Multiplex PCR amplifications employed three markers: (i) chuA, (ii) yjaA, and (iii) TSPE4.C2. Isolates were classified as belonging to one of the four phylogenetic groups A, B1, B2, or D by use of a dichotomous decision tree [22, 23]. Pyelonephritic isolate J96, human fecal isolate JJ055, and canine UTI isolate L31 were used as positive controls for phylogroups B2, D, and A, respectively.

Virulence genotyping

*E. coli* isolates were tested for the presence of ten virulence genes of various functional categories using a multiplex PCR assay with appropriate positive and negative controls. VF studied were: adhesins papA (P fimbrial structural subunit), papG alleles I, II (P fimbrial adhesin variants), fimH (type 1 fimbriae), toxins hlyA (hemolysin) siderophores: chuA (heme binding protein); yfcV (encodes major subunit of a putative chaperone- usher fimbria) capsule synthesis specific for group II (K1, K5, K12, etc.) kpsMII; serum resistance‐associated traT, upaH. J96 pyelonephritis isolate, 2H25 urosepsis isolate, V27 urosepsis isolate, L31 canine UTI isolate, and 2H16 urosepsis isolate were used as positive controls while human faecal isolate JJ055 was used as negative control [24,25].

UPEC control strains used in the current study were kindly provided by JR Johnson, VA Medical Centre and the University of Minnesota, Minneapolis, USA. The virulence p elements (papA, papGI, and papGII) were considered as a single pap factor. Thus, if a strain was positive for at least one or more of the studied pap markers, it was regarded as pap‐positive and given a score of 1. The results of such in vitro testing predict experimental virulence in vivo.

Data analysis and interpretation

Frequency and percentages of ESBL carbapenem-resistant *E. coli* were calculated. Comparisons of proportions of given characteristics between CA UTI and HA UTI population were done by Pearson χ2 test or Fisher exact test. A p-value of <0.05 was considered significant. All analysis was carried out using the SPSS version 20.0 statistical software package (IBM Inc., Armonk, New York).

## Results

A total of 150 *E. coli* isolates (CA=100; HA=50) were analyzed for antimicrobial susceptibility patterns, phylogroups, and 10 putative virulence-associated genes in a tertiary care hospital in northern India. 

Antibiotic susceptibility profile

**Figure 1:**
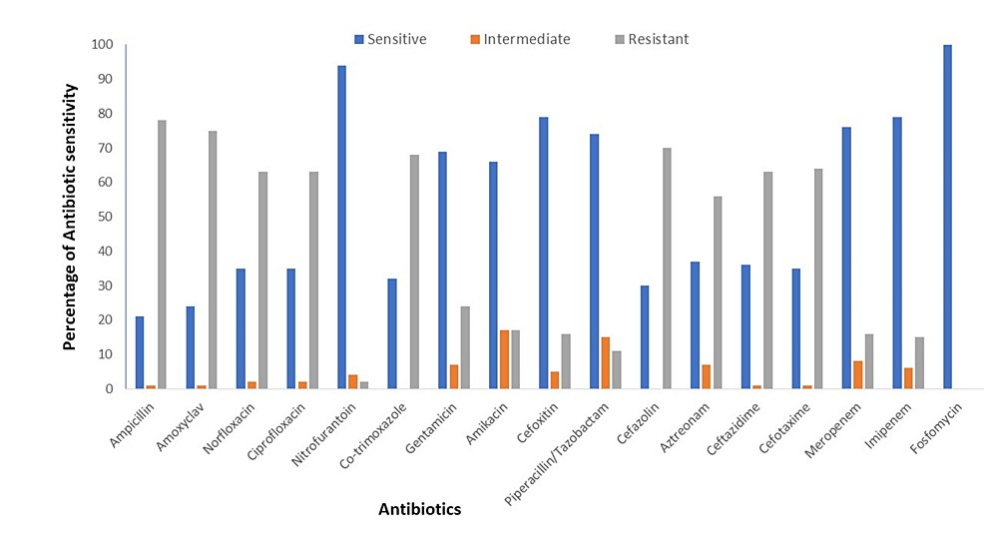
Antibiotic sensitivity profile of E. coli isolates associated with community-acquired UTI E. Coli - Escherichia coli

**Figure 2:**
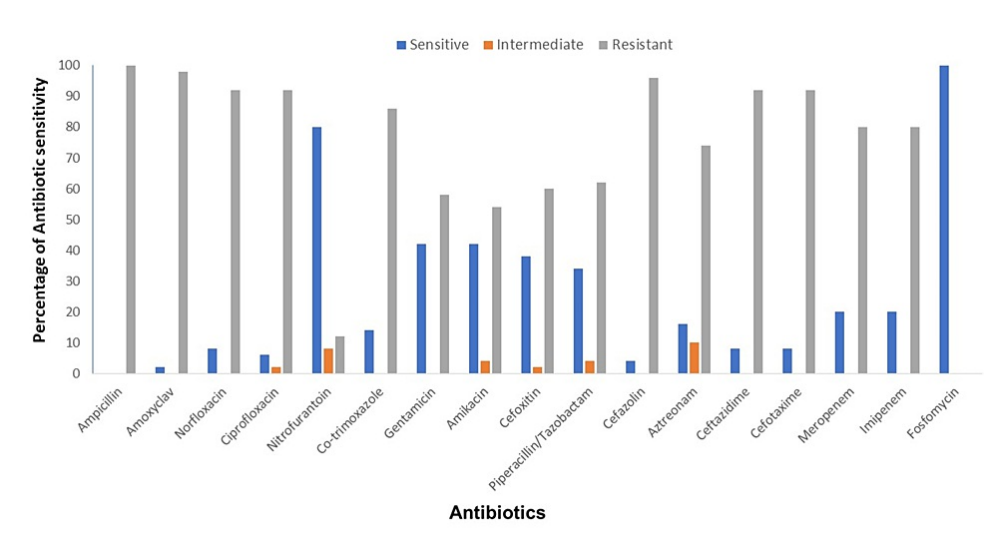
Antibiotic sensitivity profile of E. coli isolates associated with hospital-acquired UTI E. coli - Escherichia coli

*E. coli* isolates associated with HA UTI were more drug-resistant than that of CA UTI (Figure 1, 2). Among both the groups, ampicillin was the most resistant drug (HA=100% & CA=78%).

More than 90% of *E. coli* isolates associated with HA UTI were resistant to ceftazidime, cefotaxime, ciprofloxacin, and norfloxacin, while among the CA group, the resistance pattern was ceftazidime (63%), cefotaxime (64%), norfloxacin and ciprofloxacin (63%). Sixty-eight percent of CA UTI isolates were resistant to cotrimoxazole, whereas among the HA UTI isolates, resistance was 86%. Eighty percentof HA UTI isolates were resistant to carbapenem while it was only 16% among *E. coli* isolates associated with CA UTI.

Only 78% of the isolates were multidrug resistant (MDR) among CA UTI isolates but it was 100 % in the case of HA UTI isolates. Overall HA UTI *E. coli* isolates were significantly associated with resistance to almost all antibiotics (p<0.05; Table 1).

**Table 1:** Antibiotic susceptibility pattern of E. coli isolates in percentage which belonged to pathotypic and commensal phylogroups of CA UTI and HA UTI *E. coli - Escherichia coli*, CA - community-acquired, HA *-* hospital-acquired Note: p-value shown in the table are calculated by comparing antibiotic-resistant profiles of two groups of *E.coli* isolates which are associated with CA UTI and HA UTI

Antimicrobial agent	CA UTI *E. coli* isolates (N=100)		HA UTI *E. coli* isolates (N=50)		p-value
	Pathotypic group (B2 and D; N=66), n (%)	Commensal group (A and B1; N=34), n (%)	Pathotypic group (B2 and D; N=24), n (%)	Commensal group (A and B1; N=26), n (%)	
Ampicillin	50 (75.8)	28 (82.4)	24 (100)	26 (100)	0.001
Amoxyclav	49 (74.2)	26 (76.5)	23 (95.8)	26 (100)	0.001
Norfloxacin	42 (63.6)	21 (61.8)	23 (95.8)	23 (88.5)	0.001
Ciprofloxacin	41 (62.1)	22 (64.7)	23 (95.8)	23 (88.5)	0.001
Nitrofurantoin	2 (3)	0 (0)	3 (12.5)	3 (11.5)	0.017
Co-trimoxazole	42 (63.6)	26 (76.5)	19 (79.2)	24 (92.3)	0.001
Gentamicin	17 (25.8)	7 (20.6)	14 (58.3)	15 (57.7)	0.001
Amikacin	15 (22.7)	2 (5.9)	13 (54.2)	14 (53.8)	0.001
Cefoxitin	10 (15.1)	6 (17.7)	12 (50)	18 (69.2)	0.001
Piperacillin/tazobactam	7 (10.6)	4 (11.8)	13 (54.2)	18 (69.2)	0.001
Cefazolin	46 (69.7)	24 (70.6)	23 (95.8)	25 (96.2)	0.001
Aztreonam	37 (56)	19 (55.9)	20 (83.3)	17 (65.4)	0.032
Ceftazidime	41 (62.1)	22 (64.7)	22 (91.6)	24 (92.3)	0.001
Cefotaxime	41 (62.1)	23 (67.7)	22 (91.6)	24 (92.3)	0.001
Meropenem	11 (16.7)	5 (14.7)	18 (75)	22 (84.6)	0.001
Imipenem	12 (18.1)	3 (8.8)	18 (75)	22 (84.6)	0.001

Among CA UTI isolates, 54% of *E. coli* strains were positive for ESBL production whereas only 50% of HA UTI isolates were positive for ESBL production phenotypically. Multidrug resistance, ESBL production, and carbapenem resistance were significantly distributed among HA UTI isolates (p<0.05).

Distribution of phylogroups among CA and HA cystitis isolates

Based on the results obtained from triplex PCR, *E.coli* isolates were divided into four phylogroups A, B1, B2, and D. Phylogroups B2 and D belong to the pathogenic strain of *E. coli,* while phylogroups A and B1 belong to the commensal group. Fifty-two percent of *E. coli* isolates associated with HA UTI belong to commensal phylogroups A and B1 in comparison to that of the CA UTI group (34%). Among *E. coli* isolates associated with CA UTI, group B2 (34%) was dominant followed by group D (32%). In *E. coli* isolates associated with HA UTI, group A (38%) was found dominant followed by B2 (30%). Overall pathogenic phylogroups were predominant in CA *E. coli* isolates (p<0.05).

Virulence score was calculated for each isolate as the number of virulence genes detected. The average virulence score was higher for CA UTI isolates (4.25) than for HA UTI (3.9). It was also seen that most of the VFs were more commonly present in CA UTI isolates except for traT and yfc, which were higher in frequency amongst the HA UTI group (p<0.05, Table 2).

**Table 2:** Distribution of virulence factors between E. coli isolates associated with community-acquired UTI and hospital-acquired UTI *E. coli - Escherichia coli,* CA - community-acquired, HA *-* hospital-acquired

	HA UTI E.coli (N=50), n (%)	CA UTI E.coli (N=100), n (%)	p-value
papA	10 (20)	33 (33)	0.097
fimH	26 (52)	57 (57)	0.561
papGI	0	2 (2)	0.553
kpsMTII	13 (26)	59 (59)	0.001
hlyA	0	10 (10)	0.031
papGII	17 (34)	42 (42)	0.344
traT	35 (70)	58 (58)	0.153
chuA	23 (46)	66 (66)	0.019
upaH	10 (20)	55 (55)	0.001
yfc	20 (40)	20 (20)	0.009

Adhesin genes papA (p=0.097), papGI (p=0.553), papGII (0.344), and fimH (0.561) were equally distributed among both groups. The toxin-producing gene (hlyA) and iron-chelating gene (chuA) were more frequently found among *E. coli* isolates associated with CA UTI (Table 2).

Most of the VFs were equally distributed amongst MDR and non-MDR strains except for fimH (0.004) and traT (0.007), which were significantly associated with MDR strains (Table 3).

**Table 3:** Distribution of virulence factors among the MDR and non-MDR groups of E. coli isolates *E. coli - Escherichia coli*, MDR - multidrug resistant, non-MDR - non multidrug resistant

	MDR (N=128), n (%)	Non-MDR (N=22), n (%)	p-value
papA	39 (30.5)	4 (18.2)	0.239
fimH	77 (60.1)	6 (27.3)	0.004
papGI	1 (0.78)	1 (4.5)	0.273
kpsMTII	60 (46.9)	12 (54.5)	0.506
hlyA	9 (7)	1 (4.5)	1
papGII	54 (42.2)	5 (22.7)	0.084
traT	85 (66.4)	8 (36.4)	0.007
chuA	75 (58.6)	14 (63.6)	0.656
upaH	53 (41.4)	12 (54.5)	0.251

## Discussion

Urinary tract infection is one of the most common bacterial infections, and the emergence of multidrug resistance among *E. coli* isolates associated with UTIs has become a public health concern worldwide. We studied antimicrobial susceptibility profile, phylogenetic grouping, and various virulence-associated traits and their correlation among *E. coli* isolates associated with community-acquired UTI (n=100 and hospital-acquired UTI (n=50) in the current study. On detailed analysis, we found that majority (66%) of CA isolates were from pathotypic phylogroups, i.e., B2 and D, and had a higher average virulence score (4.25) than HA strains (3.9). HA *E. coli* isolates were significantly more drug-resistant than CA isolates, and MDR HA *E. coli* isolates had a significant association with some putative virulence factors like fimH, traT, and yfc. 

Resistance to some commonly used antibiotics, such as norfloxacin (92% vs. 63%), cefotaxime (92% vs. 64%), and trimethoprim-sulphamethoxazole (86% vs. 68%), was significantly higher among HA isolates as compared to that of CA UTI isolates. Our results were consistent with the concept that isolates obtained from hospital-acquired infections are more resistant than community-acquired infections due to selection pressure, erratic use of antibiotics, etc. [26]. Our results were in accordance with many previous studies where hospital-acquired isolates were more drug-resistant than community-acquired isolates [27].

Significantly higher resistance to carbapenem was found among HA UTIs isolates than CA UTIs (80% vs. 16%). A high frequency of antibiotic resistance among UPEC strains was reported in previous studies in Iran [26, 28, 29]. Studies from other countries also reported a high frequency of resistance to antibiotics in *E. coli *strains isolated from urine samples [28, 30, 31, 32]. Around 78% isolates of CA UTI and 100% isolates of HA UTI demonstrated multidrug resistance phenotype in our study. Multidrug resistance was higher in our study while many studies have reported variable frequency of MDR ranging from 15%-97% [33, 34]. This is a well-known fact that susceptibility patterns may vary from region to region, as antibiotic susceptibility depends upon the availability and utilization of antibiotics, host factors, etc. [35, 36].

Based on phylogenetic analysis, *E. coli* isolates associated with HA UTI were more frequently associated with commensal phylogroups (52%) as compared to that of CA UTI, where pathotypic phylogroups (B2 and D; 66%) were more predominant than commensal phylogroups (A and B1; 34%). The pathotypic phylogenetic group was significantly higher in CA *E. coli* isolates than in HA *E. coli* isolates. Our results were in agreement with the previous studies in which phylogroups B2 and D were more frequent in the community-acquired group [36]. Surface virulence factors such as adhesins are required by bacteria to get colonized in urinary tract. Adhesin genes papA (33% vs. 20%), fimH (57% vs. 54%), papGI (2% vs. 0), and papGII (42% vs. 34%) were found more frequently among CA *E. coli* isolates than HA *E. coli*. Many studies have reported variable frequency (0%-77%) of various adhesin genes among *E. coli* isolates [36-38]. The frequency of the fimH gene was about 73.4% (CA *E. coli*) and 67.7% (HA *E. coli* isolates), while pap genes were about 40-50% among UPEC. Though they were not significantly associated, adhesin genes, including , were more frequently found among CA *E. coli* isolates. This can be explained as UPEC strains utilize a variety of adhesins to bind successfully to urinary epithelial cells so as to initiate infection; the adherence potency of CA *E.coli* isolates was greater than that in HA isolates due to these genes [26].

VFs encoding serum resistance protein protects bacteria from the lethal activity of serum; thus, the traT gene is important for UPEC to protect them from the adverse environment of the urinary tract. traT was more frequently present in HA UTI isolates (70%) than CA isolates (58%). Also, this gene was significantly associated with multidrug-resistant organisms (p<0.008). Many studies have shown that 70-77% of cystitis *E. coli* isolates contain the traT gene, and around 76% of multidrug isolates were associated with the presence of the traT gene. Kudhina et al. in their study have shown that traT was present in more than 70% isolates [39]. Our results were comparable with many studies which suggest that virulence factor traT can be further studied as a target for therapeutic options [39, 40].

We found significant association of kpsMTII (p=0; 59% vs. 26%), hlyA (p=0.033; 10% vs. 0%), chuA (p=0.029; 66% vs. 46%) and upaH (p=0; 66% vs. 46%) among CA UTI isolates as compared with HA UTI isolates. Though there are very few studies available that have studied these genes specifically for comparing any difference in VFs repertoires among HA and CA UTI isolates. Few studies have reported the presence of these genes more frequently in HA UTI isolates [36]. Among all the VFs studied, only yfcV was present more significantly distributed among HA UTI isolates (p=0.009; 40% vs. 20%) in the present study. This result was in agreement with a previous study where a high frequency of yfcV was found among *E. coli* isolates associated with HA infection. 𝛼-hemolysin is an important virulence factor encoded by hlyA gene, which mediates the release of iron from red blood cells, and causes dysfunctioning of phagocytic activity and cytotoxic damage to the cells [41].

Many studies have reported that hlyA, chuA, iron uptake genes, etc. are present much more frequently among isolates associated with outpatients than inpatients UTIs [42]. A study done in Mexico revealed that most of the *E. coli* isolates associated with CA UTI had kpsMII, which is responsible for the formation of the capsule. The fimH virulence gene was also present in around 61% of isolates [34].

We found that many MDR isolates were frequently associated with P-fimbriae (papA), fimH, traT, yfcV, etc., of which fimH, traT, and yfcV were significantly associated with multidrug-resistant isolates. Ochoa et al. have found that many MDR-UPEC isolates had high positivity for the presence of fimH, an iron uptake gene (chuA), and a toxin gene (hlyA) [33]. It has been postulated that virulence genes may share the same loci with some antibiotic resistance-producing genes and may transmit themselves from one bacteria to another.

By analyzing our data, we observed that HA *E. coli* isolates had a high frequency of multidrug resistance and comparatively reduced VFs content. It has been suggested that some of the VFs can be used as potential targets for anti-infective therapies or the development of a vaccine [43]. As we observed that there was a reduced prevalence of VFs among HA isolates, using them as a target for therapy could be more challenging [44]. 

## Conclusions

As evident from the present study, the majority of *E. coli* associated with CA cases belongs to pathotypic phylogroups B2 & D and possess a variety of virulence genes that promote efficient colonization of the urinary tract. VGs like fimH, papA, kpsMII, fyuA, traT, and afa/draBC were more frequently present in CA *E. coli* as opposed to HA isolates. For an *E. coli* to cause CA UTI, it needs to be more virulent, whereas a lesser virulent *E. coli* can cause HA UTI. Hence, it can be apparently assumed that a strain with lesser virulence is able to cause health care associated urinary tract infection, as compared to community-acquired UTI, probably indicating that immune status/general condition of the patients is an important determinant in acquiring infection rather than virulence potential of pathogen alone. MDR was present in every HA *E. coli* isolate, and fimH, traT, and yfc genes showed significant association with MDR strains. This aspect can further be explored to study for comprehensive research focusing on new therapeutic medicines and vaccines against these putative virulence factors.
